# Ibrutinib-Induced Perianal Rash Complicated by Bacterial Infection in a Patient With Chronic Lymphocytic Leukemia: A Case Report and Literature Review

**DOI:** 10.7759/cureus.82952

**Published:** 2025-04-24

**Authors:** Jugraj Singh, Palak Gupta, Juan Vargas, Andrzej Kudelka, Margaret Kobe, Neychelle Rocca

**Affiliations:** 1 Internal Medicine, Northern Arizona Healthcare, Cottonwood, USA; 2 Internal Medicine, Maharishi Markandeshwar Institute of Medical Sciences and Research, Ambala, IND; 3 Hematology and Oncology, Northern Arizona Healthcare, Cottonwood, USA; 4 Infectious Disease, Northern Arizona Healthcare, Cottonwood, USA

**Keywords:** chronic lymphocytic leukemia, cutaneous adverse effects, ibrutinib, perianal cellulitis, perianal rash

## Abstract

Ibrutinib, a Bruton's tyrosine kinase (BTK) inhibitor, is widely used in the treatment of chronic lymphocytic leukemia (CLL). However, its use is associated with a range of adverse effects, including cutaneous manifestations. We present a case of a 74-year-old male with CLL who developed a perianal rash following four years of ibrutinib therapy. The rash progressively worsened due to a superimposed bacterial infection, resulting in perianal cellulitis with bleeding, severe pain, and difficulty passing stools. The patient was started on antibiotics, and ibrutinib was discontinued. This case highlights an unusual presentation of ibrutinib-induced skin toxicity in the perianal region, complicated by bacterial infection, and underscores the importance of early recognition and management. To the best of our knowledge, this is the first report of such a manifestation, contributing new insights into the spectrum of ibrutinib-associated cutaneous adverse effects.

## Introduction

Chronic lymphocytic leukemia (CLL) is a monoclonal lymphoproliferative disease characterized by the increased production of mature but dysfunctional B lymphocytes. It is the most common adult leukemia in Western populations, comprising 25-30% of leukemias in the United States [1]. Bruton's tyrosine kinase (BTK) is a key component of the B-cell receptor signaling pathway, which mediates the proliferation, survival, migration, and homing of both normal and malignant B cells [2]. Ibrutinib, a BTK inhibitor, provides a significant overall survival benefit and progression-free survival in CLL by irreversibly inhibiting the BTK pathway, leading to a significant reduction in survival and proliferation of malignant B cells [3,4].

Although ibrutinib is widely used in the treatment of CLL and other B-cell malignancies, cutaneous manifestations have been reported in 2-27% of patients. The most common cutaneous adverse effects include rashes, petechiae, and bruising. Two distinct types of rash have been reported: one is a nonpalpable, largely asymptomatic petechial rash, while the other is a palpable, pruritic, nonblanching, violaceous papular eruption resembling leukocytoclastic vasculitis. Classified using the Common Terminology Criteria for Adverse Events (CTCAE) version 5.0, these rashes are typically mild (grade I-II); however, severe allergic drug reactions, such as lip tingling and tongue swelling, have been observed in patients with grade III rashes [5].

We report a case of an elderly patient with CLL who developed a perianal rash following four years of ibrutinib therapy. The rash worsened due to a superimposed bacterial infection, leading to perianal cellulitis with bleeding and significant tenderness. This ultimately necessitated the discontinuation of ibrutinib therapy.

## Case presentation

The patient is a 74-year-old male who presented to the emergency department with complaints of a perianal rash that had been present for the past five months. His symptoms worsened in the past week, with severe pain, bleeding, and an inability to pass stools due to the pain. He denied any abdominal pain, fever, chills, or constipation. He had no history of diabetes mellitus or any other comorbidities.

**Figure 1:**
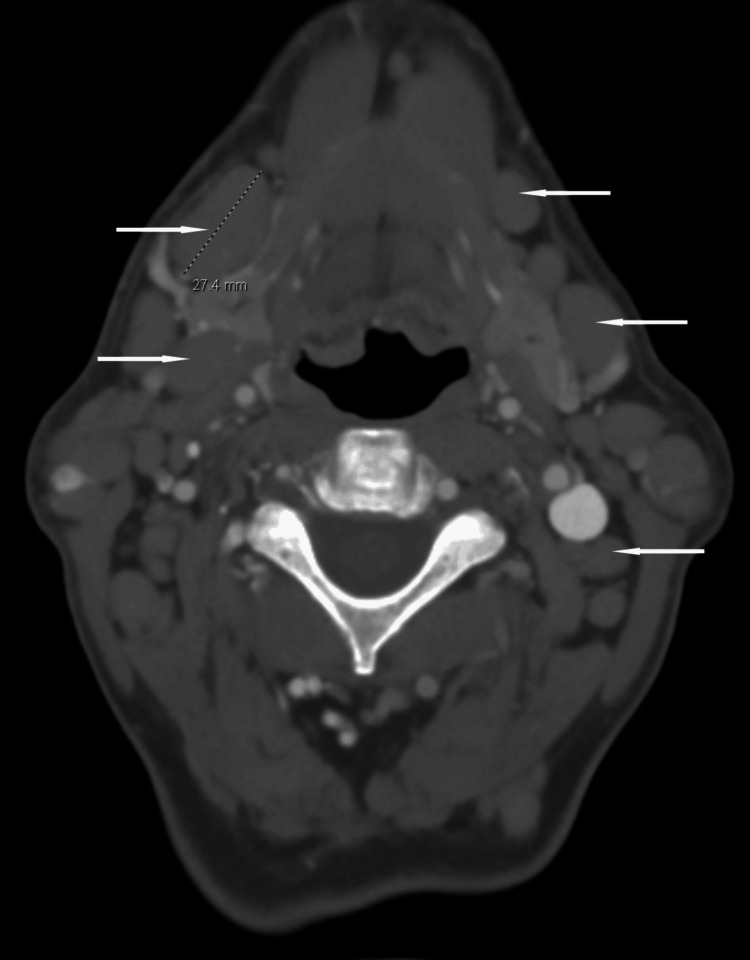
Contrast-enhanced computed tomography scan (axial view) of the head and neck showing enlarged cervical lymph nodes (arrows).

**Figure 2:**
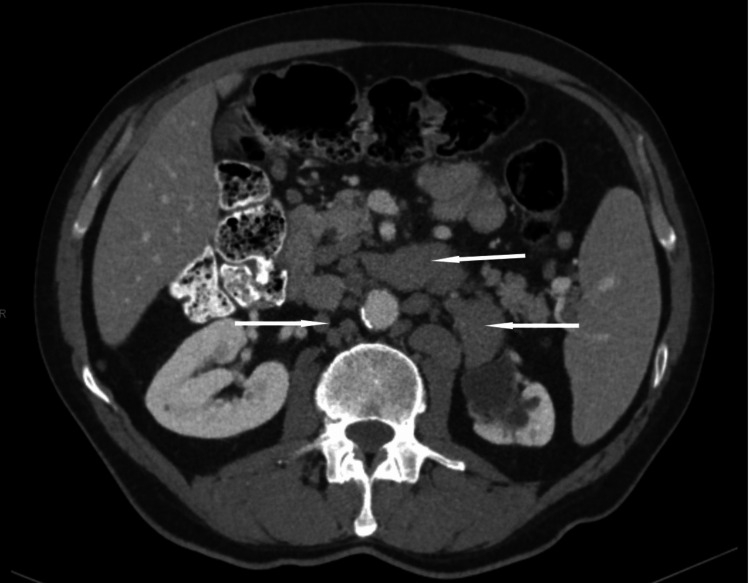
Contrast-enhanced computed tomography scan of the abdomen showing enlarged para-aortic and mesenteric lymph nodes (arrows).

The patient was diagnosed with CLL in 2016 and developed extensive adenopathy. A contrast-enhanced computed tomography (CECT) scan of the head and neck revealed severe adenopathy, with involvement of the cervical lymph nodes (Figure 1). Similarly, a CECT scan of the abdomen showed extensive, diffuse adenopathy affecting the para-aortic and mesenteric lymph nodes (Figure 2). A left cervical lymph node biopsy confirmed CLL/small lymphocytic leukemia (SLL), with a bone marrow biopsy showing extensive involvement. Peripheral blood was morphologically normal, but flow cytometry showed monoclonal kappa light chain restriction, and fluorescence in situ hybridization (FISH) analysis revealed trisomy 12. The patient received six cycles of bendamustine and rituximab with an excellent response. In 2020, biopsies of inguinal lymph nodes and nasal sinus tissues confirmed persistent CLL/SLL, with nasal endoscopy revealing the involvement of ethmoid and maxillary sinuses. He was started on ibrutinib 420 mg daily.

**Figure 3:**
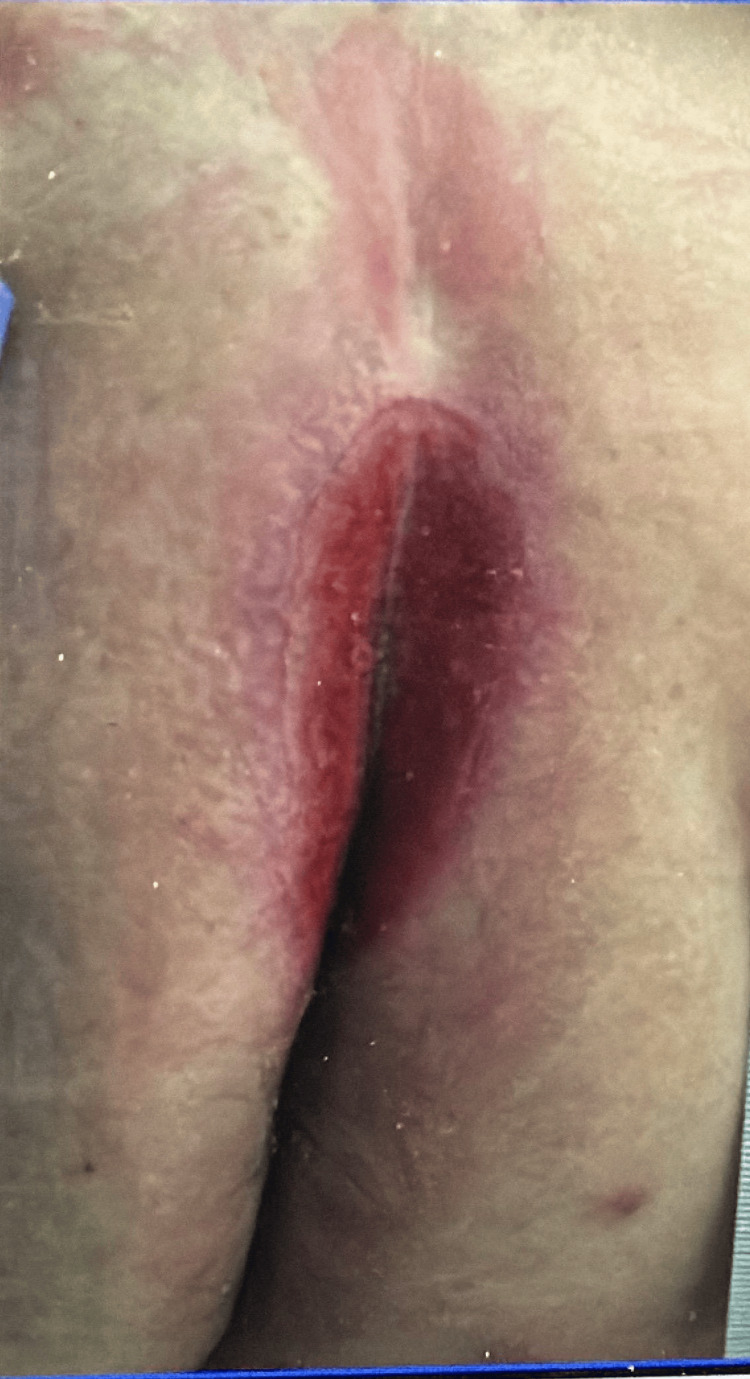
Perianal rash having a superficial burn-like appearance.

On this visit, the patient was hemodynamically stable, with a blood pressure of 142/72 mmHg, a heart rate of 65 beats/minute, a respiratory rate of 18 breaths/minute, and an oxygen saturation of 97% on room air. Physical examination revealed a rash localized to the perianal region, characterized by a superficial, burn-like appearance, along with extensive ulcerations, discharge, and bleeding. The area was extremely tender to touch (Figure 3). Laboratory results showed a white blood cell count of 6.5 × 10^3^/μL, C-reactive protein (CRP) of 10.2 mg/L, and a lactic acid level of 0.9 mmol/L, suggesting mild systemic inflammation. Blood cultures were negative, while wound cultures from the perianal region identified *Staphylococcus aureus* and *Pseudomonas *species.

The patient was started on empiric broad-spectrum antibiotics (vancomycin and cefepime), and ibrutinib was discontinued. Labs were repeated, and the lymphocyte count remained normal. Wound care was consulted, and conservative wound care was recommended, with no need for surgery. The broad-spectrum antibiotics were discontinued, and the patient was switched to amoxicillin-clavulanate (Augmentin) based on culture sensitivities. Morphine was prescribed for pain management. Following treatment with antibiotics and discontinuation of ibrutinib, the rash showed improvement. A diagnosis of perianal rash secondary to ibrutinib, complicated by a superimposed bacterial infection leading to perianal cellulitis, was made. A decision was made to start alternative antineoplastic therapy on an outpatient basis based on the lymphocyte count and the patient's symptoms related to CLL.

## Discussion

CLL affects over 200,000 individuals and leads to approximately 4,410 deaths annually in the United States [6]. CLL is associated with a weakened immune system and an increased risk of infection-related complications [6]. Diagnosis is typically established through blood counts, blood smears (with an absolute lymphocyte count of greater than 5,000/mcL and smudge cells), and immunophenotyping of circulating B-lymphocytes, which identify a clonal B-cell population expressing the CD5 antigen along with typical B-cell markers [7].

Symptoms of CLL can range from asymptomatic to severe complications. For asymptomatic patients, regardless of disease risk category, clinical observation is typically recommended. However, patients with symptomatic disease, such as lymphadenopathy, hepatosplenomegaly, or those with low neutrophil counts, anemia, thrombocytopenia, and/or B symptoms like fever, drenching night sweats, and weight loss, should be offered treatment. A regimen using either a B-cell leukemia/lymphoma 2 (BCL2) inhibitor (venetoclax) or a covalent BTK inhibitor (acalabrutinib, zanubrutinib, or ibrutinib) is considered first-line therapy. There is no evidence that commencing one type of drug before the other leads to better results. With survival rates of roughly 88% at four years for acalabrutinib, 94% at two years for zanubrutinib, and 78% at seven years for ibrutinib, covalent BTK inhibitors are usually administered indefinitely [6].

Ibrutinib is associated with several side effects, the most common being diarrhea, upper respiratory tract infections, bleeding, fatigue, and cardiac issues [5]. The most frequent causes of death associated with ibrutinib-containing regimens, aside from CLL, include unexplained or unwitnessed death, infection, and secondary cancers [5]. Ibrutinib and other BTK inhibitors are also known to cause various cutaneous manifestations, including cellulitis, neutrophilic dermatoses, ecthyma, lichenoid eruption, onychomadesis, paronychia, pyogenic granuloma (periungual), pityriasis rosacea, and pyoderma gangrenosum.

The most common cutaneous side effects are rashes, petechiae, and bruising. Ibrutinib-induced rashes have been reported in patients with mantle cell lymphoma (MCL), chronic myelogenous leukemia (CML), and CLL who are taking 400-600 mg of the medication. Rash onset can vary, with some patients experiencing symptoms as late as 300-400 days after beginning treatment [8]. The pathophysiology behind cutaneous reactions is likely related to the off-target inhibition of the epidermal growth factor receptor (EGFR), a mechanism shared by other tyrosine kinase inhibitors. EGFR inhibition can lead to skin eruptions by promoting apoptosis, inflammation, and disruption of cell cycle progression. Another proposed mechanism involves the inhibition of c-kit and platelet-derived growth factor receptors [9].

Our patient, who was diagnosed with CLL and started on ibrutinib, developed a perianal rash following four years of treatment. To date, case reports, mostly from Western populations, have described ibrutinib-induced skin rashes affecting areas such as the nape of the neck, trunk, axilla, and limbs. To the best of our knowledge, this is the first report of ibrutinib causing skin toxicity in the perianal area, which led to a superimposed bacterial infection causing perianal cellulitis that required ibrutinib discontinuation and treatment with antibiotics.

## Conclusions

This case underscores the potential for ibrutinib to cause unusual cutaneous adverse effects, such as a perianal rash, which may become complicated by superimposed bacterial infections. Clinicians should be vigilant in monitoring for skin reactions, especially in areas prone to moisture and friction, as these may predispose to infections. Early recognition and prompt management, including discontinuation of ibrutinib and appropriate antibiotic therapy, are crucial in preventing severe complications. Given the rarity of perianal involvement, this case contributes to a broader understanding of ibrutinib-related dermatologic toxicities and highlights the need for individualized care when managing patients with CLL on BTK inhibitor therapy.
